# Efficacy and Radiographic Analysis of Minimally Invasive Posterior Mono-Axial Pedicle Screw Fixation in Treating Thoracolumbar Burst Fractures

**DOI:** 10.3390/jcm11030516

**Published:** 2022-01-20

**Authors:** Jae-Hoon Shim, Eun-Min Seo

**Affiliations:** Department of Orthopedic Surgery, Chuncheon Sacred Heart Hospital, Hallym University College of Medicine, Chuncheon 24253, Korea; shim0121@hallym.or.kr

**Keywords:** thoracolumbar burst fractures, mono-axial pedicle screw, poly-axial pedicle screw

## Abstract

Background: The purpose of this study was to evaluate the effectiveness of minimally invasive posterior mono-axial pedicle screws fixation in the treatment of thoracolumbar burst fractures. Methods: In the present study, we analyzed 98 patients retrospectively who had thoracolumbar burst fractures without a neurological deficit. Patients were divided into two groups: mono-axial pedicle screw fixation group (*n* = 52) and poly-axial pedicle screw fixation group (*n* = 46). We collected clinical data (visual analog scale (VAS) score for back pain) and included radiographic measurements. Results: Sagittal index was significantly improved at postop and last follow-up in the mono group and the poly group. The mono group was better for reducing and maintaining anterior vertebral height. For the mono group, the mean postoperative regional kyphosis correction rate was 62.31%, and correction loss was 14.18% in late follow-up. For the poly group, the mean postoperative regional kyphosis correction rate was 52.17%, and correction loss was 33.42% in late follow-up. The mono-axial pedicle screw group had a good correction rate and reduced the risks of correction loss. The mean VAS scores for back pain improved by 2.4/2.5 and 3.8/4.2 for the mono and poly groups, respectively. There was no significant difference between groups. Conclusions: The mono-axial pedicle screw fixation was better for reducing and maintaining anterior vertebral height and regional kyphosis. Therefore, the mono-axial pedicle screw is a better optional instrumentation to treat thoracolumbar vertebral fractures.

## 1. Introduction

The most common spinal fractures have been reported to be in the thoracolumbar region [1] because it is in the transition zone between the rigid thoracic kyphosis and more flexible lumbar lordosis [2].

Conservative treatment is usually the method of choice if there is little kyphotic deformity, no neurological deficit or no unstable posterior vertebral column [3,4,5]. In unstable thoracolumbar burst fractures, operative stabilization is preferred [6,7,8,9]. The main goals of surgery are restoration of spinal stability, correction of deformity, and decompression of the spinal canal with the preservation of neurologic function [10,11,12]. An anterior or posterior approach, or combined approaches are used for the treatment of thoracolumbar burst fractures [13,14]. However, there is no consensus regarding the best surgical approach. Nevertheless, spine surgeons often prefer the posterior approach because of its easy application, reduction of intraoperative bleeding and low degree of invasiveness [11]. It has become the most used method in the surgical treatment of thoracolumbar burst fractures [15,16]. Thus, a transpedicular screw is popularly used to treat thoracolumbar burst fractures. Many biomechanical studies demonstrate that the designs of the screw head play a significant role in the correction of spinal deformity and have different effects on the stiffness in three dimensions (coronal, sagittal, and axial plane) [17].

According to biomechanical theory, the mono-axial pedicle screw could, more effectively than the poly-axial pedicle screws, restore the vertebral height, correct the kyphosis, reduce the postoperative kyphosis loss, and maintain fracture reduction. However, the question arose to whether the mono-axial pedicle screw fixation could really achieve vertebral height recovery, improve kyphosis, reduce postoperative kyphosis loss, and reduce the incidence of internal fixation failure better than the poly-axial pedicle screw fixation in clinical practice.

Hence, we decided to compare and analyze the clinical and radiological results after posterior fixation by mono-axial pedicle screws and poly-axial pedicle screws in thoracolumbar burst fractures.

## 2. Materials and Methods

### 2.1. Patients

From January 2013 to January 2020, 287 patients with thoracolumbar spine fractures received surgical treatment in our institution. To obtain retrospective follow-up results, we selected from these patients a group of patients according to the following inclusion criteria: a burst-compression injury at T12–L1 that involved the middle column but left the posterior column intact, no neurological deficit, and no pathologic fracture. Based on AO thoracolumbar classification system, all patients were the A3 fracture type. Exclusion criteria were pathologic or osteoporotic fracture or a history of previous back surgery.

We selected 98 patients and divided them into 2 groups: mono-axial pedicle screw fixation group (*n* = 52) and poly-axial pedicle screw fixation group (*n* = 46). Mono- and poly-axial pedicle screws were used throughout the study period. Poly-axial pedicle screws were mainly used in the early study period and in the less severe fractures. Mono-axial pedicle screws were frequently used in order to achieve better fracture reduction. The male to female ratio was 2.27:1, with 68 males and 30 females patients. The majority of fractures resulted from falling down. All patients underwent plain X-rays, computed tomography (CT) and magnetic resonance imaging (MRI) before surgery. The most fractured sites were L1 fractures in 63 (64%) and T12 in 35 (36%). Follow-up times ranged from 18 to 43 months. The mean age was 49.3 years in the mono group and 52.9 years in the poly group. Surgery of the fracture was performed on average 2.9 days after hospital admission. Posterior screw fixation involved 3 levels (fracture level and above and below level of the fracture vertebra) in both groups. Demographic data are presented in Table 1.

We collected clinical data (visual analog scale (VAS) score for back pain) and included radiographic measurements. The latter were used to calculate sagittal plane kyphosis.

The study adhered to the principles of the Declaration of Helsinki with voluntary participation. All experiments were carried out in accordance with relevant guidelines and regulations. Informed consent was obtained from all participants. The data were handled on a group level, and personal details were replaced by identification codes. The research was approved by the Chunchon Sacred Heart Hospital, Hallym University College of Medicine Investigational Review Board (IRB) for the Protection of Human Subjects.

### 2.2. Operative Technique

To minimize the influence of surgical technique on outcomes, one senior surgeon performed all operations. Patients were positioned prone on the four poster frames in order to reduce the intra-abdominal pressure and to create a positional reduction effect on the fracture. A minimally invasive posterior para-median approach was used to minimize iatrogenic tissue injury in all patients. We used 6.5 mm diameter titanium mono- and poly-axial pedicle screws (Mega spine system, JOYM Co., Ltd., Seoul, Korea). All screw replacements were confirmed by radiographs. All procedures were the same in both groups, except for screw type. 

In the group, mono-axial pedicle screws were inserted parallel to the superior end plate of the upper and lower adjacent vertebra. Then, intermediate screws were inserted into the pedicles of the fractured vertebrae parallel to the upper endplate of the upper adjacent normal vertebrae. Fracture reduction and indirect decompression of the spinal cord were achieved by applying distraction and producing an appropriate contour in the rod. Details of the reduction procedure were as follows: Temporarily tightening of the screw nuts with pre-contoured rods on mono-axial pedicle screws, which were inserted parallel to the superior end plate, forming a 90°–90° screw–rod construct. This led to reduction of the anterior vertebral height (AVH) and segmental kyphosis of the fractured vertebrae. Indirect reduction of the posterior vertebral height (PVH) was performed via ligamentotaxis after connecting rods distraction cranially and caudally in sequence. Final tightening of the whole construct was performed. The length of the spine was restored. 

In the poly group, poly-axial pedicle screws were inserted parallel to the superior end plate of the upper and lower adjacent vertebra. Then, intermediate screws were inserted into the pedicles of the fractured vertebrae parallel to the upper endplate of the upper adjacent normal vertebrae. The rods were fixed, and then torque was applied through the rod pusher to bring the vertebrae back to the rod. Details of the reduction procedure were the same in both groups. 

Bracing was prescribed for patients for 8 weeks after surgery. The purpose of bracing was to prevent implant failure and pseudarthrosis through limit of motion. Implant removal was performed at 1 to 2 years after surgery when the bone healed. 

### 2.3. Radiologic Evaluation and Clinical Assessments

**Figure 1:**
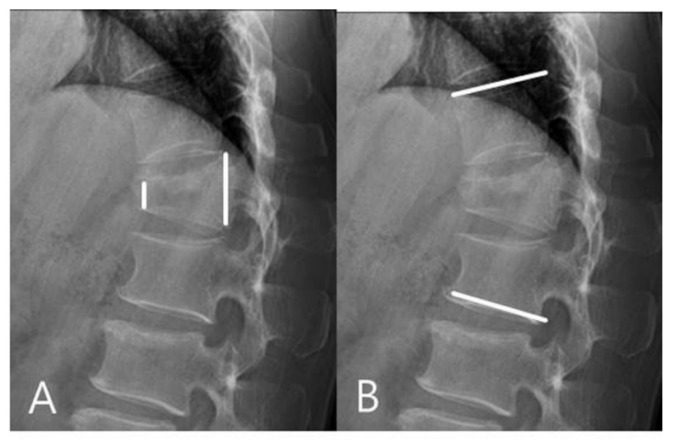
(**A**) Sagittal index (SI) was calculated by the ratio of the heights of the anterior and posterior vertebral wall. (**B**) Regional kyphosis angle (RKA) was measured on lateral radiographs using the Cobb method. RKA between the superior endplate of the vertebra above the fractured vertebra and the inferior endplate of the vertebra below the fractured vertebra were measured.

Radiographic evaluations were based on anteroposterior (AP) and lateral views, flexion and extension lateral views, and three-dimensional CT scans. Using the ratio of the heights of the anterior and posterior vertebral wall (on lateral views of the injured vertebral body), we calculated the sagittal index (SI) preoperatively, immediately after surgery, and at the final follow-up (Figure 1). Kyphotic deformity was evaluated on lateral views using the Cobb method. Regional kyphosis angle (RKA) between the superior endplate of the vertebra above the apical (injured) vertebra and the inferior endplate of the apical vertebra below were measured preoperatively, immediately after surgery, and at the final follow-up (Figure 1). 

**Figure 2:**
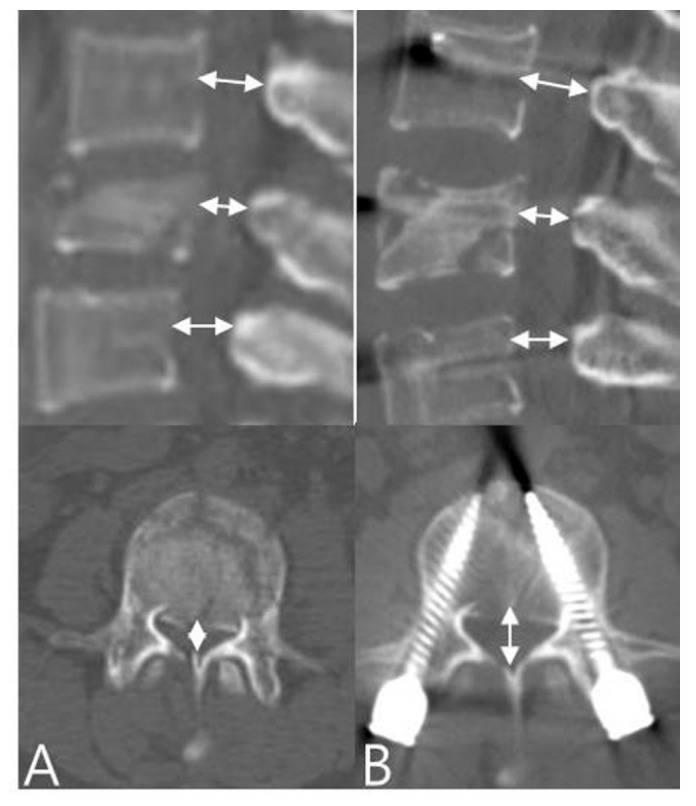
Spinal canal encroachment and clearance were calculated (**A**) before and (**B**) after sur gery on CT scans. The percentage of canal compromise was calculated with the narrowest mid-sagittal diameter of the injured level divided by the mean of the mid-sagittal diameters of the adjacent upper and lower vertebrae.

RKA does not only stand for the deformity of the fractured vertebral body, but also the destruction of the affected intervertebral disc. Spinal canal encroachment and clearance were calculated before and after surgery on CT scans. The percentage of canal compromise was calculated with the narrowest mid-sagittal diameter of the injured level divided by the mean of the mid-sagittal diameters of adjacent upper and lower vertebra (Figure 2) [5,18].

Surgical time, operative blood loss, and perioperative complications were assessed. For clinical outcomes, assessment was analyzed using a VAS for the back: Oswestry Disability Index (ODI).

### 2.4. Statistical Analysis 

The PACS system (π view^®^, Infinitt, Seoul, Korea) was used by 2 independent observers for the measurement. The intraobserver and interobserver agreement rate and k values were obtained to check errors between 2 observers. For statistical analysis, the SPSS 22.0 was used, and *p* values less than 0.05 were considered significant. Continuous variables are presented as means ± SD. Frequency analysis was used for categorical variables. ANOVA and the Kruskal–Wallis test were used as appropriate for group comparisons.

## 3. Results

### 3.1. Radiographic Outcomes

The interobserver agreement rate was 94% (mean k = 0.75), and the intraobserver agreement rate was 97% (mean k = 0.81). The intraobserver and interobserver error analyses showed good agreement.

The radiographic outcomes are summarized in Table 2. 

For the mono group, the preoperative mean SI was O.59 ± 0.12 (range, 0.37–0.77), the postoperative SI was O.80 ± 0.09 (range, 0.62–0.98), and the last follow-up SI was O.76 ± 0.09 (range, 0.56–0.89). For the poly group, the preoperative SI was O.57 ± 0.11 (range, 0.34–0.82), the postoperative SI was O.73 ± 0.11 (range, 0.46–0.91) and the last follow-up SI was O.65 ± 0.11 (range, 0.36–0.87). The SI was significantly improved at postop and last follow-up in the mono group and poly groups. The mono group was better for reducing and maintaining anterior vertebral height.

**Figure 3:**
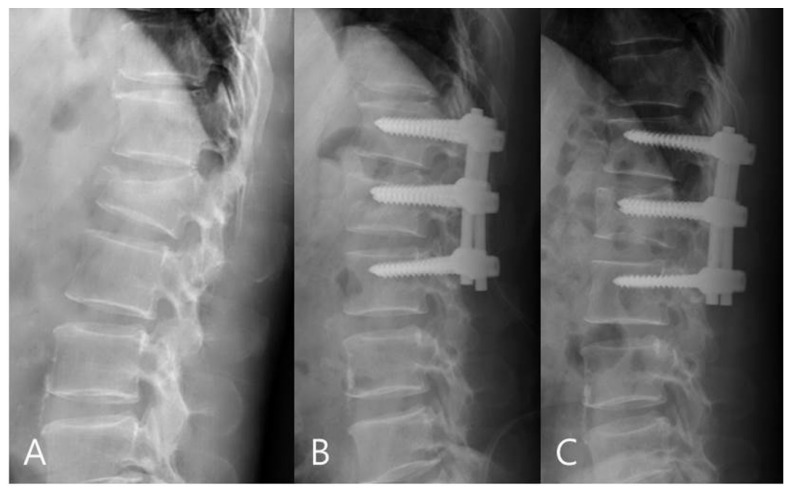
(**A**) 58-year-old male patient with L1 burst fracture was treated by mono-axial pedicle screw fixation. (**B**) Postoperative and (**C**) final follow-up imaging show good correction and no correction loss.

**Figure 4:**
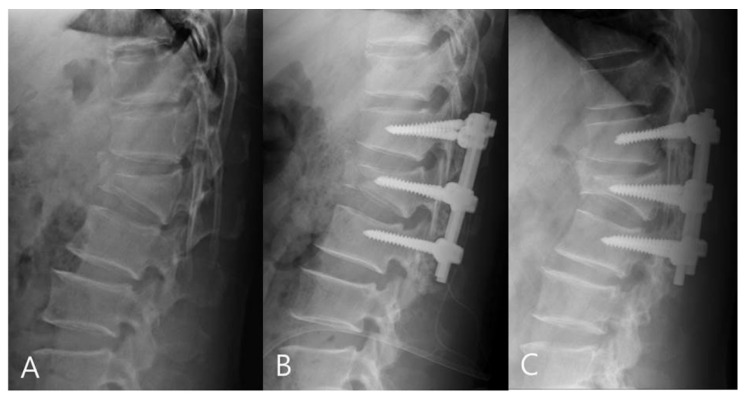
(**A**) 45-year-old male patient with L1 burst fracture was treated by poly-axial pedicle screw fixation. (**B**) Postoperative imaging showed a mild correction of anterior body height and kyphosis. (**C**) Final follow-up imaging showed correction loss.

For the mono group, the preoperative mean RKA was 21.56°, and the postoperative mean RKA was 8.13°. The mean correction angle was 13.43° (correction rate 62.31%). The RKA angle decreased from 8.13° to 11.18° (correction loss: 14.18%) in late follow-up. For the poly group, the preoperative mean RKA was 23.18°, and the postoperative mean sagittal plane kyphosis was 11.09°. The mean correction angle was 12.09° (correction rate 52.17%).The RKA decreased from 11.09° to 18.83° (correction loss: 33.42%) in late follow-up. The mono-axial pedicle screw fixation had a better correction rate and reduced the risks of correction loss versus the poly-axial pedicle screw fixation (Figure 3 and Figure 4).

The mean preoperative canal narrowing was 38.6%, which was recovered 14.8% and 15.1% after surgery and at the last follow-up, in the mono group. The mean preoperative canal narrowing was 36.4%, which was recovered 22.3% and 23.7% after surgery and at the last follow-up, in the poly group. The mean preoperative canal narrowing was improved in both groups, and a statistical difference was found between both groups (*p* < 0.05). An improvement of spinal canal encroachment in the mono group was better than that found in the poly group. 

Bone fusion was obtained at the final follow-up of both groups, and there was no difference in fusion rate.

### 3.2. Clinical Outcomes

Mean blood loss was 120 mL (range, 50–200 mL) in the mono group and 110 mL (range, 40–180 mL) in the poly group. The mean operative time was 55 min (range, 35–135 min) in the mono group and 60 min (range, 40 –125 min) in the poly group (Table 3). 

The mean VAS scores for back pain improved from 6.4 preoperatively to 2.1 at the last follow-up (*p* < 0.05) in the mono group, and from 6.5 preoperatively to 2.2 at the last follow-up (*p* < 0.05) in the poly group. There was no significant difference between groups with regard to VAS scores.

The number of complications were similar between the two groups (Table 4). 

There were five cases of screw pull-out in the mono group. Three cases out of them were developed during the intraoperative reduction procedure. There were four cases of screw pull-out in the poly group. One case out of them was developed during the intraoperative reduction procedure. The superficial wound infection in the mono group was successfully treated using debridement, primary closure over drains, and antibiotic therapy. The case with rod fracture in the mono group required revision of the hardware. There were no severe complications such as neurological deficits in both groups.

## 4. Discussion

The goals of surgical treatment for thoracolumbar burst fractures are to restore vertebral body height, correct angular deformity, decompress neural tissue, allow rapid mobilization and rehabilitation, prevent development of progressive deformity with neurologic deficit, and limit the number of instrumented vertebral motion segments [19,20,21,22]. In recent years, with advancements in surgical techniques and instrumentation, minimally invasive surgical techniques have been introduced. In particular, the minimally invasive posterior pedicle screws fixation using percutaneous or para-median mini-open approach is one of the good options in the treatment of thoracolumbar fractures. Many authors had compared clinical and radiological outcomes between minimally invasive surgical techniques and open surgical techniques and had reported similar clinical and radiological outcomes [19,22].

The ideal surgical management remains controversial, and no evidence-based guideline for the most optimal surgical approach or instrumentation technique has been developed [23,24,25]. Long segment pedicle screw fixation may be stiffer and may impart greater forces on adjacent segments compared with short segment fixation, which may affect clinical performance and long-term outcome but at the cost of sacrificing additional motion segments [26]. For this reason, short-segment posterior fixation (one level above and below the fracture level) has been used more commonly than long-segment posterior fixation for the treatment of thoracolumbar burst fractures [11,27,28]. However, some studies showed that short segment posterior fixation alone led to a 9–54% incidence of implant failure and rekyphosis at long-term follow-up, and 50% of the patients with implant failure had moderate to severe pain [29]. It is important to obtain the best fracture reduction as possible [30]. The greater residual kyphotic deformity provides greater anterior vertebral stress on pedicle screws. Thus, the overloading force on the instrument loosens the screw, causing it to break, dislodge, and disconnect, which is mostly seen in short-segment posterior fixation [26,29,30]. To overcome this situation, some studies suggested that pedicle screws be added to the fracture level [31] because the stiffness increased an average of 160% when using the additional pedicle screw fixation at the fracture level. Axial testing showed that the six-screw construct was 84% stiffer than the four-screw construct in flexion and was 38% stiffer than the four-screw construct in torsional testing [15]. The additional pedicle screw fixation at the fracture level can function as a push point with an anterior vector, creating a lordorizing force that restored anterior vertebral height and the segmental kyphosis. Therefore, short-segment posterior fixation with pedicle screw fixation at the fracture level provided better anterior vertebral height restoration and kyphosis correction for thoracolumbar burst fractures [23].

Many biomechanical studies demonstrated that the designs of the screw head play a significant role in the correction of spinal deformity and have different effects on the stiffness in three dimensions (coronal, sagittal, and axial plane) [17]. An experimental study reported by Wang showed that the mono-axial pedicle screw with no motion between the screw head and shaft can significantly increase the stiffness in the axial direction compared with the poly-axial pedicle screw and can reduce the risks of correction loss [32]. Mono-axial pedicle screws can be a better fixation instrumentation for thoracolumbar burst fractures in theory. However, the question arose to whether the mono-axial pedicle screw fixation could really achieve vertebral height recovery, improve kyphosis, reduce postoperative kyphosis loss, and reduce the incidence of internal fixation failure better than the poly-axial pedicle screw fixation in clinical practice. Hence, we planned this study.

In this study, our results found that there was a significant difference in the reduction of anterior vertebral height and correction of the kyphosis angle (sagittal plane kyphosis) between the mono group and poly group. The mono group was better for reducing and maintaining anterior vertebral height and reducing the kyphosis angle because, the mono-axial pedicle screw with no motion between the screw head and shaft formed a 90°–90° screw–rod construct. However, the poly-axial pedicle screw with motion between the screw head and shaft could not form a 90°–90° screw–rod construct. This led to a significant difference in the reduction of anterior vertebral height and correction of the kyphosis angle (sagittal plane kyphosis) between the mono group and poly group.

Therefore, minimally invasive posterior mono-axial screw fixation could provide sufficient immobilization to restore spine stability until the fracture healed, thus obtaining satisfactory reduction and maintenance of the fractured vertebrae height.

In this study, there were five cases of screw pull-out in the mono group. Three cases out of them were developed during the intraoperative reduction procedure. Specially, over-contoured rods can lead to screw pull-out in old patients with osteoporosis. Thus, it requires attention when pre-contoured rods are connected on mono-axial pedicle screws, and connecting rods are distracted cranially and caudally. In addition, using cement-augmented pedicle screws could be considered.

This study has some limitations. First, this is a retrospective study. Second, 98 patients is a small group for such a clinical study. Third, there was a short follow-up time and the adjacent intervertebral space height was not taken into account; therefore, the results may be biased.

## 5. Conclusions

The mono-axial pedicle screw fixation was better for reducing and maintaining anterior vertebral height and reducing the kyphosis angle. Therefore, mono-axial pedicle screw fixation may provide sufficient immobilization to restore spine stability until the fracture heals, thus obtaining satisfactory reduction and maintenance of the fractured vertebrae height.

## Figures and Tables

**Table 1:** Summary of demographic profile.

Demographics	All Patients	Mono Group	Poly Group	*p* Value
No. of patients	98	52	46	0.755
Age (years)	50.1 ± 13.4	49.3 ± 14.8	52.9 ± 12.6	0.063
Gender (M:F)	2.27:1	2.47:1	2.07:1	0.323
Follow-up (months) (mean ± SD)	33.3 ± 15.1	27.9 ± 14.6	34.7 ± 16.7	0.626
Timing from trauma to surgery (day)	2.9 ± 0.8	2.8 ± 0.9	3.1 ± 0.7	0.761
Level of fracture (*n* (%))				
T12	35(36)	20(38)	15(33)	0.622
L1	63(64)	32(62)	31(67)	0.634

**Table 2:** Comparisons of radiographic data between the two groups.

	Mono Group	Poly Group	*p* Value
Regional kyphosis angle (degrees) (°)			
Preoperation	21.56° ± 8.5°	23.18° ± 7.6°	0.865
Postoperation	8.13° ± 5.8°	11.09° ± 7.6°	<0.05
Final follow-up	11.18° ± 6.4°	18.83° ± 8.5°	<0.05
*p* value (pre-final)	<0.05	<0.05	
Sagittal index (SI)			
Preoperation	O.59 ± 0.12	O.57 ± 0.11	0.863
Postoperation	O.80 ± 0.09	O.73 ± 0.11	<0.05
Final follow-up	O.76 ± 0.09	O.65 ± 0.11	<0.05
*p* value (pre-final)	<0.05	<0.05	
Spinal canal encroachment (%)			
Preoperation	38.6 ± 11.4	36.4 ± 17.6	0.412
Postoperation	14.8 ± 9.7	22.3 ± 10.3	<0.05
Final follow-up	15.1 ± 10.8	23.7 ± 11.6	<0.05
*p* value (pre-final)	<0.05	<0.05	

**Table 3:** Comparisons of clinical outcomes between the two groups.

	Mono Group	POLY Group	*p* Value
Operation time(min) Blood loss(mL) VAS back score	55.1 ± 57.6 120.3 ± 82.9	60.0 ± 44.4 110.6 ± 69.6	0.756 0.471
Preoperation	6.4 ± 3.5	6.5 ± 3.3	0.33
Postoperation	2.2 ± 1.4	2.2 ± 1.2	0.47
Final follow-up	2.1 ± 1.6	2.2 ± 1.4	0.83
*p* value (pre-final)	<0.001	<0.001	

**Table 4:** Summary of complications.

	Mono Group	Poly Group
Implant failure	7 (13%)	4 (9%)
screw fracture	1 (2%)	0 (0%)
Screw pullout	5 (9%)	4 (9%)
rod fracture	1 (2%)	0 (0%)
Pseudarthrosis	0 (0%)	0 (0%)
Perioperative complications		
Infection	1 (2%)	0 (0%)
Neurological deficit	0 (0%)	0 (0%)

## Data Availability

All relevant data are within the manuscript.
